# Pertactin-Deficient *Bordetella pertussis*, Vaccine-Driven Evolution, and Reemergence of Pertussis

**DOI:** 10.3201/eid2706.203850

**Published:** 2021-06

**Authors:** Longhuan Ma, Amanda Caulfield, Kalyan K. Dewan, Eric T. Harvill

**Affiliations:** University of Georgia College of Veterinary Medicine, Athens, Georgia, USA

**Keywords:** Bordetella pertussis, bacteria, pertussis, whooping cough, vaccines, acellular vaccine, whole-cell vaccine, pertactin, PRN, antibody titers, waning immunity, reemergence, pertactin deficient, vaccine-driven evolution, respiratory infections, United States

## Abstract

Recent reemergence of pertussis (whooping cough) in highly vaccinated populations and rapid expansion of *Bordetella pertussis* strains lacking pertactin (PRN), a common acellular vaccine antigen, have raised the specter of vaccine-driven evolution and potential return of what was once the major killer of children. The discovery that most circulating *B. pertussis* strains in the United States have acquired new and independent disruptive mutations in PRN is compelling evidence of strong selective pressure. However, the other 4 antigens included in acellular vaccines do not appear to be selected against so rapidly. We consider 3 aspects of PRN that distinguish it from other vaccine antigens, which might, individually or collectively, explain why only this antigen is being precipitously eliminated. An understanding of the increase in PRN-deficient strains should provide useful information for the current search for new protective antigens and provide broader lessons for the design of improved subunit vaccines.

*Bordetella pertussis,* the causative agent of pertussis (whooping cough), continues to reemerge in countries that have high vaccine coverage, such as the United States, and has accelerated since the switch during the mid-1990s from whole-cell pertussis (wP) formulations comprising many partially characterized bacterial proteins to the less reactogenic 1–5 component acellular pertussis (aP) vaccines (*1**,**2*). These aP vaccines, including DTaP (diphtheria, tetanus, and aP for children) and Tdap (tetanus, diphtheria, and aP for adolescents and adults), protect against disease, but this protection wanes rapidly and does not prevent colonization or transmission of the pathogen (*3**–**5*). In this background of suboptimally performing aP vaccines, many countries have noted the emergence and expansion of strains specifically lacking pertactin (PRN), a membrane bound autotransporter, and 1 of up to 5 *B. pertussis* protein antigens included in the vaccines (*6**–**11*).

PRN-deficient *B. pertussis* strains have recently been reported in countries using aP vaccines, including the United States, Australia, Sweden, Italy, Norway, the United Kingdom, France, Belgium, Finland, the Netherlands, and Japan. The frequency of PRN-deficient strains has been variable, but these strains have risen to dominance in the United States (85%), Australia (>80%), Sweden (69%), and Italy (55%) (*7**–**11*). Lower frequencies were reported from Japan, which showed a major decrease from a prevalence of 41% during 2008–2010 to 8% during 2014–2016 and correlated with a change to aP vaccine formulations that exclude PRN (*6*,*11**–**12*). Denmark, which uses the monocomponent pertussis toxin (PT) vaccine, had no reports of PRN-deficient isolates before 2012, and the 4 PRN-deficient strains detected since have been associated with human migration from countries with PRN in their vaccines (*6*). Limited data are available from the predominantly developing countries that use wP vaccines to enable a robust comparison between the effects that aP and wP have on the selection of PRN. A sequencing study of the only 2 clinical isolates reported from India, which still uses wP, showed that the isolates still retained the broadly encountered PRN gene allele *prn-1* (*13*). Considered together, these observations provide a strong correlation between the use of aP vaccines containing PRN and the appearance and increase to prominence of PRN-deficient strains.

Lineages of all bacteria are constantly evolving, but increasing to dominance alone is not conclusive evidence of a causal relationship between use of PRN-containing aP vaccines and loss of PRN. A PRN mutation could be carried along with a strain that is increasing in dominance because of 1 or many other mutations. However, the appearance of a wide variety of PRN mutations, each arising from a diversity of *B. pertussis* lineages over time, provides additional strong evidence in favor of vaccine-driven selection on PRN in particular. Although insertions of IS481 at multiple genomic locations are the most common PRN mutation, there is a large diversity of disruptions to PRN expression, including deletions within the signal sequence, promoter inversion, transversions resulting in a stop codon, deletions resulting in a stop codon, and full-gene deletion (*2*,*6*,*12*). The variety of genomic lesions that have led to loss of PRN indicates that numerous independent selection and expansion events have occurred in most of the lineages now circulating in many countries using aP vaccines. Providing more direct experimental data, such as murine models aimed at investigating PRN-deficient *B. pertussis* infection, have demonstrated an overall defect in colonization in unvaccinated mice, but advantages in both colonization and competition in assays using aP-vaccinated mice (*14**–**16*).

Together, these observations strongly support the hypothesis that loss of PRN confers a fitness advantage over wildtype *B. pertussis* particular to the aP vaccinated populations in which they are arising. However, there are 4 other antigens included in aP vaccines that are not being disrupted or lost. Why are the other vaccine antigens not being mutated at similar rates? What are the characteristics of PRN that might lead to the loss of this antigen in particular? Understanding multiple possible explanations, and distinguishing between them where possible, will be useful for ongoing efforts to improve vaccines to control *B. pertussis* spread and disease.

## Role of PRN

PRN is an autotransporter protein located on the surface of *B. pertussis* (*17*). Similar to all autotransporters found in gram-negative pathogens, PRN has 3 functional domains: the N terminal signal sequence, the passenger domain, and a C-terminal autotransporter domain. The signal sequence guides the passenger and transporter domains into the periplasm, enabling the transporter domain to form a pore in the outer membrane for translocation of the passenger domain to the cell surface. The protein is then cleaved by an outer membrane protease, the passenger domain remaining in contact with the surface by noncovalent interactions (*18**,**19*).

The function of PRN is only partially understood. PRN is considered one of several virulence factors found in *B. pertussis* and has been shown to serve as an adhesin, facilitating attachment to various mammalian epithelial cells (*20**,**21*). The 3-dimensional structure of PRN (PDB no. 1DAB) shows 16 right-handed parallel β-helixes, the largest β-helix structure recorded to date. Two Arg–Gly–Asp tripeptide motifs within the helical structure appear to be potential attachment sites to many mammalian adhesion proteins (*22**–**24*). PRN is reported to be essential for resisting neutrophil-mediated clearance and possesses additional immunomodulatory abilities that aid *B. pertussis* in suppressing the production of proinflammatory cytokines (*25**,**26*). The benefits of functional expression of PRN in pathogenesis are consistent with its conservation in *B. pertussis* and other pathogenic *Bordetella* species. Loss of such a factor would be expected to be costly to the organism, yet PRN-deficient strains appear to be rapidly expanding in aP vaccinated populations, suggesting a recent rebalancing of fitness costs and benefits.

## Unique Features of PRN among Vaccine Antigens

PRN is conserved in *B. pertussis*, *B. parapertussis*, and *B. bronchiseptica* under the same *Bordetella* virulence gene regulatory system, suggesting that PRN has a more general role in pathogenesis that is not restricted to the human-specific *B. pertussis*. However, there are 16 other autotransporter genes identified in the genome of *B. pertussis*, 5 of which are disrupted by frameshift mutations (*27*). Park et al. (*28*) observed that autotransporters, as a group, are much more highly mutated or lost than other virulence factors. There appears to be no major loss in in vitro growth or in vivo fitness that prevents the expansion of *B. pertussis* strains lacking PRN, indicating that the functional contributions of PRN in pathogenesis might be redundant or that complementary functions, potentially mediated by other autotransporters, might be compensating for any deficiencies caused its loss. This finding can be contrasted with PT, which requires a complex operon to assemble and another to export, has a central and nonredundant role in the pathogenesis of *B. pertussis*, and has no paralogs in the genome that can replace it. This finding is consistent with the rarity of clinical isolates lacking PT, compared with the abundance of PRN-deficient strains (*29**–**31*). For these and potentially other reasons, it might be that loss of PRN can be tolerated, but loss of PT would result in a more serious fitness defect.

**Figure 1:**
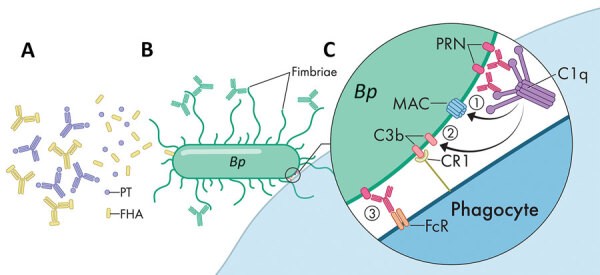
Model for various roles of antibodies against antigens in acellular pertussis vaccine. A) Antibodies against PT and FHA neutralize secreted virulence factors and mitigate disease progression but are not targeted to the bacterial surface. B) Antibodies attaching to fimbriae poorly activate the complement system far from the bacterial membrane. C) Antibody-PRN complex induces strong bactericidal activity via multiple synergistic functions. This complex activates complement to form a MAC, activates complement to deposit components such as C3b that opsonize the bacterial surface, and binds FcRs on phagocytes to activate phagocytosis. PRN labels indicate strains specifically lacking PRN. *Bp*, *Bordetella pertussis*; CR1, complement receptor type 1; FcR, fragment crystallizable region; FHA, filamentous hemagglutinin; MAC, membrane attack complex; PRN, pertactin; PT, pertussis toxin.

Examination of the location and conformation of the different acellular vaccine components showed that most antibody functions directed against these antigens occur away from the bacterial surface. Both PT and the mature surface-associated filamentous hemagglutinin (FHA) (*32*) are secreted and diffuse away into the surrounding host environment. Therefore, antibodies directed against released FHA and PT would primarily neutralize toxin function by binding to the secreted molecules in the surrounding milieu, or to molecules in the process of being shed, mitigating potential surface-directed antibody effects. These antibodies do not effectively localize to the bacterial surface and thus do not facilitate complement activation or fragment crystallizable region (FcR)–mediated phagocytosis that would result in direct bacterial killing (Figure 1, panel A) (*33*,*34*). Fimbriae (FIM), although generally anchored to the bacteria, might extend more than a cell length away from the cell surface. Because FIM are composed of repeated structures with many copies of the same antigenic molecule, most FIM antibodies would be bound to parts of the structure extended >1 micron from the bacterial membrane, which is an enormous distance for highly reactive complement components to travel. Antibodies bound to fimbria would not be arrayed on a 2-dimensional surface required to optimally bind and activate complement or FcRs (Figure 1, panel B) (*35*).

However, PRN is the only aP vaccine antigen that remains closely associated with the outer membrane. When cognate antibodies bind PRN, they become arrayed on the bacterial surface in a conformation that is particularly effective in binding and activating complement component C1q. The subsequent complement cascade that is activated rapidly deposits component in the adjacent membrane that opsonizes the cell and assembles into a membrane attack complex to lyse the bacterial membrane (*33**–**36*). The combination of the array of antibodies, bolstered by the complement components cleaved and activated in the immediate vicinity, would effectively opsonize the bacteria for efficient phagocytic killing (Figure 1, panel C).

The model we provide (Figure 1), although largely hypothetical, is consistent with evidence that aP vaccination is effective in preventing severe disease (by binding and neutralizing a key factor that mediate aspects of disease) but is much less effective in preventing nasopharyngeal colonization. Most antibodies, similar to those directed against secreted PT/FHA or distal FIM, do not effectively target and kill the bacteria. This model would also explain why loss of PRN might enable partial evasion of aP-induced immunity, and is also consistent with human surveys that showed that 3-component aP vaccines containing PT, FHA, and PRN were more efficacious than 2-component vaccines lacking PRN (*37*,*38*).

## Persistence of PRN Antibodies

**Figure 2:**
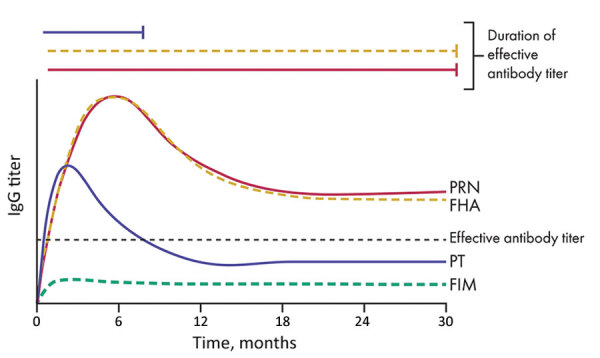
Differential decay of antibodies against acellular pertussis vaccine antigens and their effective capacity for protection. Antibodies against PRN and FHA remain at relatively higher titers for a longer period. However, PT-specific antibodies decrease to low titers rapidly. A consistently low level of antibodies against FIM is induced. Solid lines indicate antibodies that have high protective capacity, and dotted lines indicate antibodies that had low protective capacity. Only PRN antibodies are highly protective and persist at high titers for years. FHA, filamentous hemagglutinin, FIM, fimbriae; PT, pertussis toxin; PRN, pertactin.

Despite the shortcomings of aP vaccines in generating a strong memory response, and the resulting waning immunity of these vaccines against disease (*39**,**40*), aP vaccines induce robust IgG titers against most of its component antigens. However, this strong humoral response, although protecting against symptoms of pertussis, does not prevent the pathogen from colonizing the upper respiratory tract or from transmitting between hosts (*3*). Furthermore, although initially induced at high levels, circulating antibodies decay relatively rapidly across all age groups (*4**,**40**–**43*). Studies evaluating dynamic levels of specific antibodies across time have consistently show differential rates of decay for the various antigen-specific antibodies, with PT antibody titers decaying more rapidly than antibodies to FHA and PRN (Figure 2). Similarly, FIM2/3 have been reported to poorly stimulate generation of protective antibodies postinfection and postvaccinations (*4**,**43*), although these data conflict with those of other reports that shown higher levels of FIM antibodies, even at 10 years postvaccination. However, the higher antibody titers observed in these reports are also noted to decrease sharply and are likely to reach unprotective levels over a relatively short time. This rapid waning immunity against PT and FIM would be expected to narrow the window of selective pressure against these antigens.

In contrast to antibodies against PT, antibodies against PRN and FHA are relatively more persistent (Figure 2) (*38**,**44**,**45*), suggesting that there is a longer period after vaccination when there are effective titers of antibodies against FHA and PRN. This finding would be expected to result in strong pressure against PRN and FHA. However, in a search for serologic correlates of immunity to pertussis, Le et al. (*4*) noted that FHA provides relatively little contribution to protection but PRN had a higher protective role.

These observations have recently been validated in studies by Lesne et al., who used human serum bactericidal assays to determine that antibodies to PRN, but no other aP component, are bactericidal in in vitro complement killing assays (*46*). These findings somewhat conflict with those of previous studies and testing methods, which often prioritize PT IgG as an indicator for protection against pertussis (*4**,**45**,**47*). However, the short period during which levels of neutralizing antibodies against PT remain elevated, in contrast to bactericidal antibodies against PRN, suggests that PRN antibodies might be a more appropriate measure for pertussis immunity.

The short period during which antibodies to PT remain at elevated levels indicates that there is a longer period when antibodies to PRN remain at high levels, but levels of antibodies to PT have decreased. Le et al. also noted that a much larger proportion of enrolled patients tested before aP vaccination already had high titers of antibodies to PRN, and many had antibodies to PT (*4*). Therefore, in addition to its surface localization and strong opsonizing potential, the persistence of the PRN antibodies is likely to contribute to prolonged selection against this antigen in particular.

## Discussion

The rapid reemergence of pertussis noted in the early 2000s brought much initial speculation. Factors such as human migration, increased sensitivity of testing, increased volumes of testing, and reporting through heightened surveillance have been proposed to contribute to the observed resurgence (*1**–**3*). However, the collective experience in countries switching from wP vaccines to aP vaccines strongly suggests that these safer, but less effective, vaccines have contributed to the resurgence of pertussis. In addition, the way multiple PRN-deficient strains have swept across countries that use aP vaccines presents a strong case in favor of vaccine-driven selection against PRN (*6**–**12*). What has remained unclear is what will be the consequences of these changes. Will PRN-deficient strains continue to evolve, loosing other vaccine antigens until they escape vaccine effects completely? Or will the loss of these factors result in strains attenuated in virulence such that they become more like commensals than pathogens? Or is PRN really the only antigen that can be lost without serious fitness costs?

The 3 aspects of PRN we have highlighted for selective loss of PRN (i.e., its functional redundancy, the relatively longer functional persistence of antibodies against it, and its close location to the surface membrane for productive complement fixation) are not an exhaustive list of all possibilities and are not mutually exclusive. Sufficient sustained selective pressure against any particular antigen is likely to lead to its loss or change. Efforts to improve the current vaccine should be informed by a careful consideration of the lessons learned from this instance. If PRN is being lost because it is the only surface antigen, then should we replace it with 1 or more new surface antigens? If it is lost because its function is at least partly redundant then should we select some molecule(s) that is less likely to be redundant? In designing new vaccines, it would be prudent to carefully consider the issues that appear to be enabling potential vaccine escape mutants, such as PRN-deficient strains, to rapidly expand and rise to prominence.
